# Topical astilbin ameliorates imiquimod‐induced psoriasis‐like skin lesions in SKH‐1 mice via suppression dendritic cell‐Th17 inflammation axis

**DOI:** 10.1111/jcmm.17184

**Published:** 2022-01-12

**Authors:** Qingqing Xu, Zhaoyang Liu, Zhiqiang Cao, Yongjian Shi, Ning Yang, Guangshang Cao, Chunmin Zhang, Rong Sun, Chunhong Zhang

**Affiliations:** ^1^ Department of Dermato‐Venereology The Second Hospital Cheeloo College of Medicine Shandong University Jinan China; ^2^ Department of Pharmacy Affiliated Hospital of Shandong University of Traditional Chinese Medicine Jinan China; ^3^ The Second Hospital Cheeloo College of Medicine Shandong University Jinan China; ^4^ Advanced Medical Research Institute Cheeloo College of Medicine Shandong University Jinan China

**Keywords:** astilbin, dendritic cell, IL‐17‐producing T cells, keratinocyte differentiation, psoriasis

## Abstract

Astilbin, an essential component of Rhizoma smilacis glabrae, exerts significant antioxidant and anti‐inflammatory effects against various autoimmune diseases. We have previously reported that astilbin decreases proliferation and improves differentiation of HaCaT keratinocytes in a psoriatic model. The present study was designed to evaluate the potential therapeutic effects of topical administration of astilbin on an imiquimod (IMQ)‐induced psoriasis‐like murine model and to reveal their underlying mechanisms. Topical administration of astilbin at a lower dose alleviated IMQ‐induced psoriasis‐like skin lesions by inducing the differentiation of epidermal keratinocytes in mice, and the therapeutic effect was even better than that of calcipotriol. Moreover, the inflammatory skin disorder was relieved by astilbin treatment characterized by a reduction in both IL‐17‐producing T cell accumulation and psoriasis‐specific cytokine expression in skin lesions. Furthermore, we found that astilbin inhibited R837‐induced maturation and activation of bone marrow‐derived dendritic cells and decreased the expression of pro‐inflammatory cytokines by downregulating myeloid differentiation factor 88. Our findings provide the convincing evidence that lower doses of astilbin might attenuate psoriasis by interfering with the abnormal activation and differentiation of keratinocytes and accumulation of IL‐17‐producing T cells in skin lesions. Our results strongly support the pre‐clinical application of astilbin for psoriasis treatment.

## 1 INTRODUCTION

Psoriasis, an immune‐mediated inflammatory dermatosis, affects approximately 125 million people worldwide.^1^ Clinical manifestations of psoriasis include silvery white scales, slightly raised erythema and severe itching, and the histological changes include hyperproliferation and dedifferentiation of epidermal keratinocytes, increased subepidermal angiogenesis and immune cell infiltration of dendritic cells (DCs), T cells and neutrophils.^2^

As surveillance cells and antigen‐presenting cells (APCs) in the body, DCs play a central role in the pathology of psoriasis. During infection, plasmacytoid DCs (pDCs) produce large amounts of IFN‐α following the binding of single‐stranded viral RNA/DNA to the endosomal toll‐like receptor (TLR)7/9.^3^ Abundant IFN‐α regulates the maturation of inflammatory DCs (CD11c+ phenotype), the activation of which releases IL‐1β, IL‐6, IL‐12, IL‐23 and TNF‐α.^4^ Through stimulation with IL‐23 or IL‐1β, DCs drive IL‐17‐producing T cells, such as γδT cells,^5^ which produce IL‐17 and IL‐22 to induce keratinocytes to amplify inflammation and epidermal hyperplasia.^6^, ^7^, ^8^ Therefore, regulating these pro‐inflammatory cytokines in DCs would be a viable measure for psoriasis treatment.

Astilbin, an important bioactive ingredient derived from Rhizoma smilacis glabrae, is widely used in traditional Chinese medicine for the treatment of autoimmune diseases and possesses anti‐inflammatory activity and immunoregulatory effects.^9^, ^10^, ^11^, ^12^ Oral astilbin (50 or 25 mg/kg) is effective in the treatment of psoriasis by inhibiting Th17 cell differentiation.^13^ Moreover, astilbin regulated in vitro keratinocyte differentiation in our study.^14^ Previous study showed that astilbin at 50 μg/ml began to play an inhibitory role on HaCaT cell proliferation, but 50 μg/ml is a very high concentration for most compounds. Given that the DC—Th17 inflammation axis plays a central role in the pathology of psoriasis, we detected the effect of astilbin on DCs. A lower dose of astilbin at a concentration of 10–20 μg/ml can inhibit the maturation and activation of bone marrow‐derived dendritic cells (BMDCs). Therefore, in this study, we aimed to evaluate the potential therapeutic effects of topical administration of astilbin on an imiquimod (IMQ)‐induced psoriasis‐like murine model and reveal their underlying mechanisms. IMQ is a ligand of TLR7/8 and acts as a potent immune activator of DCs and causes them to mature, leading to psoriasis‐like skin lesions when applied to the skin.^15^, ^16^ IMQ‐induced skin lesions are a classic model of psoriasis. This study used the hairless, but immunocompetent SKH‐1 mice. This murine strain lacks active hair follicles and a thicker surface epidermis that closely mimics human skin. SKH‐1 mice are suitable for experimental dermatology and have been widely used in research on wound healing, atopic dermatitis and ultraviolet radiation‐induced skin photoaging.^17^, ^18^, ^19^

The results showed that topical administration of astilbin at a lower dose level (4 or 10 mg/kg daily) alleviated the IMQ‐induced psoriasis‐like skin lesions by inducing the differentiation of epidermal keratinocytes in mice. Moreover, the inflammatory skin disorder was restored by astilbin treatment characterized by reduced accumulation of both IL‐17‐producing T cells and expression of psoriasis‐specific cytokines in skin lesions via downregulation of myeloid differentiation factor 88 (MyD88). Therefore, use of astilbin to control the maturation and activation of DCs by reducing MyD88 in TLR7/8 signals may be an effective strategy to treat psoriasis.

## 2 MATERIALS AND METHODS

### 2.1 Animals

SKH‐1 hairless mice were purchased from Charles River Laboratories (Wilmington) and housed in a pathogen‐free facility at a temperature of 25 ± 5°C and 55 ± 5% humidity. The mice were given access to a standard laboratory diet and water. All animal experiments were conducted in accordance with the Guide for the Care and Use of Laboratory Animals and were approved by the Animal Care and Use Committee of the Second Hospital of Shandong University (KYLL‐2019(KJ)A‐0102).

### 2.2 Reagents and treatments

Imiquimod cream was purchased from Sichuan Med‐Shine Pharmaceutical Co., Ltd. (5%; Mingxinlidi). Calcipotriol ointment, a vitamin D analogue, was purchased from LEO Laboratories Limited (0.005%; LEO Pharma). Astilbin was purchased from Solarbio, and astilbin cream was prepared in‐house. Briefly, 8 mg or 20 mg astilbin was fully dissolved in 40 μl dimethyl sulfoxide (DMSO), 0.8 ml polyethylene glycol (PEG‐400), 0.2 ml Tween‐80 and 3 ml 2% hypromellose medium to achieve 0.2% or 0.5% astilbin respectively. Mice were treated with 60 µl ointment per 30 g of body weight, and the final dosage of astilbin was 4 or 10 mg/kg mouse daily. Ointment bases without astilbin were used as a control vehicle.

Male SKH‐1 hairless mice that were 6–8 weeks old were randomly divided into six groups (*n* = 5 per group). The control group (Con) received no IMQ cream, and the other five groups were topically administered a daily dose of 62.5 mg 5% IMQ cream on their backs for 12 consecutive days to establish IMQ‐induced psoriasis‐like skin lesions. The model group (IMQ) received IMQ cream only and the calcipotriol group received calcipotriol ointment. The astilbin‐high group (AH) received 0.5% astilbin ointment, and the astilbin‐low group (AL) received 0.2% astilbin ointment. The ointment base group (OB) received ointment without astilbin. All treatments were performed on the 6th day after IMQ was administered, once a day for 7 days. After 13 days, the mice were sacrificed by cervical dislocation under diethyl ether anaesthesia, and skin lesions and serum samples were collected.

### 2.3 Scoring severity of skin inflammation

To evaluate the severity of psoriasis‐like skin lesions, an objective scoring system was adopted based on the clinical Psoriasis Area and Severity Index (PASI). Erythema, scaling and infiltration were scored independently on a scale from 0 to 4 as follows: 0, none; 1, slight; 2, moderate; 3, marked and 4, very marked. Erythema was scored according to the red tints. The total score (erythema plus scaling plus infiltration) served as a measure of the severity of inflammation (scale from 0 to 12).

### 2.4 Skin cell preparation and flow cytometry assay

Mouse back skin was incubated in 1.2 U/ml dispase II at 4°C overnight to separate epidermis and dermis. The epidermis and dermis were incubated with trypsin‐EDTA at 37℃ for 4 h, then passed through a 200‐mesh sieve to prepare single cell suspensions. Fixation buffer, intracellular stain permeabilization, PE anti‐mouse CD3, FITC anti‐mouse CD4 and APC anti‐mouse IL‐17A mAbs were obtained from Biolegend. For intracellular staining, cells were first stained with PE anti‐mouse CD3 and FITC‐CD4 mAbs, and then fixed, permeabilized and stained with intracellularly for APC‐IL‐17A mAbs. The relevant isotype control mAbs were also used. Samples were harvested with a BD FACS Calibur and analysed using FlowJo software.

### 2.5 Skin histology and immunochemical staining

The mice were sacrificed, and the skin samples were fixed in paraformaldehyde and embedded in paraffin. Skin sections (5 μm) were stained with haematoxylin and eosin and Ki67 antibodies (Abcam), and staining was assessed using light microscopy (Olympus). Epidermal thickness was determined by measuring the average interfollicular distance under a microscope in a blinded manner.

### 2.6 Immunofluorescence staining

Skin samples from the back lesions of mice embedded and frozen in OCT medium for immunofluorescence staining. Skin sections (10 μm) were stained for KRT5 (Abcam), and staining was assessed in a blinded manner using a NanoZoomer S60 fluorescent microscope (Hamamatsu Photonics K.K.) to determine the thickness of the undifferentiated epidermis.

### 2.7 Isolation of bone marrow cells and in vitro induction and culture of BMDCs

Bone marrow‐derived dendritic cells were induced from bone marrow cells of SKH‐1 mice by flushing the femurs and tibiae of 6­ to 8‐week‐old mice with PBS. Pooled cells from the legs were treated with red blood cell lysis buffer (Solarbio) and washed with RPMI­1640 medium and cultured in six­well plates (1 × 10^7^/well) with RPMI­1640 medium supplemented with 10% foetal bovine serum (FBS) (Gibco), 1% penicillin‐streptomycin, 20 ng/ml IL‐4 and 20 ng/ml GM‐CSF (PeproTech). BMDCs were cultured for 7 days to induce DCs, and the medium was replaced daily. DCs were screened and separated using a flow cytometer for CD11c‐PE staining. On Day 8, astilbin (20 µg/ml) was added to the culture cells, and after 6 h, cells were stimulated with 1 µg/ml R837 to induce the maturation of DCs. To determine whether astilbin influences the ratio of mature and activated DCs induced by R837 in vitro, BMDCs from different groups were suspended in PBS, and then stained with anti‐CD11c‐PE (Cat. no. E‐AB‐F0991D), anti‐MHC Ⅱ‐FITC (Cat. no. E‐AB‐F0990C), anti‐CD80‐PE‐Cy.7 (Cat. no. E‐AB‐F0992H) and anti‐CD86‐APC (Cat. no. E‐AB‐F0994E) antibodies at 4℃ for 30 min in the dark. Cells were washed once with PBS and analysed with flow cytometry.

### 2.8 ELISA

Skin tissue samples from the back lesions of mice were obtained and snap‐frozen in liquid nitrogen. Then, the tissues were weighed and homogenized in ice‐cold PBS with a protease inhibitor cocktail, followed by centrifugation at 12,000 *g* for 20 min at 4°C. The supernatant was stored at −80°C and used for ELISA assays within a month.

Supernatants from cultured BMDCs stimulated with astilbin and/or R837 were collected and stored at −80°C for ELISA assays within a month. Protein levels of IL‐1β, IL‐6, IL‐17A and IL‐23 in the skin tissue samples, and culture samples were measured using ELISA kits from R&D Systems, following the manufacturer's protocol.

### 2.9 Real‐time polymerase chain reaction (RT‐PCR)

Total RNA was extracted from skin lesions using an RNAiso Plus kit (TaKaRa Bio) according to the manufacturer's instructions. cDNA was synthesized from RNA using the Prime Script™ RT reagent Kit (TaKaRa). Quantitative PCR (qPCR) analysis was performed on a real‐time PCR system (Eppendorf) using SYBR Green reaction master mix (TaKaRa). The desired gene expression levels were normalized to the level of β‐actin, and changes were calculated by the ΔΔ*C*_t_ method. The primer sequences are listed in Table 1.

**TABLE 1:** qRT‐PCR primer

Gene	Forward	Reverse
mRORrt	AACTGGCTTTCCATCATCATCTC	AAGGCGGCTTGGACCACGAT
mIL‐23a	ACCCACAAGGACTCAAGGAC	CTGCCACTGCTGACTAGAAC
mIL‐17A	CTCAGACTACCTCAACCGTTCC	GTGGTCCAGCTTTCCCTCC
mIL‐6	CTGCAAGAGACTTCCATCCAG	AGTGGTATAGACAGGTCTGTTGG
mIL‐1β	GAAATGCCACCTTTTGACAGTG	TGGATGCTCTCATCAGGACAG
mTNF‐a	CCTGTAGCCCACGTCGTAG	GGGAGTAGACAAGGTACAACCC
mβ‐actin	GTGACGTTGACATCCGTAAAGA	GCCGGACTCATCGTACTCC

### 2.10 Western blot assay

After treatment with chemicals, the cells or skin were lysed for Western blotting as described previously.^14^ The blots were incubated with primary antibodies against KRT1 (Abcam, ab83664), KRT5 (Abcam, ab52635), KRT10 (Abcam, ab76318), MyD88 (Santa Cruz Biotechnology, sc‐74532) and actin (Santa Cruz Biotechnology, sc‐8432) overnight at 4°C, followed by incubation with appropriate peroxidase‐conjugated secondary antibodies. Actin was used as an internal control. The detection system visualization (MilliporeSigma) was followed by exposure to an X‐ray film. The protein bands were then analysed using ImageJ software. The greyscale values of the detected proteins were normalized to the values of the corresponding actin to determine changes in protein expression.

### 2.11 Statistical analysis

Data were expressed as the mean ± SD or the mean ± SEM. For comparison of the significant differences between more than two groups, one‐way ANOVA tests were used, while other comparisons were performed with Student’s unpaired *t* test. All the statistical analyses were conducted using the GraphPad Prism software. Statistical significance was set at *p *< 0.05, and *p* < 0.001 was considered highly significant (***).

## 3 RESULT

### 3.1 Topical astilbin significantly attenuated IMQ‐induced psoriasis‐like skin lesions in mice in a dose‐dependent manner

**FIGURE 1:**
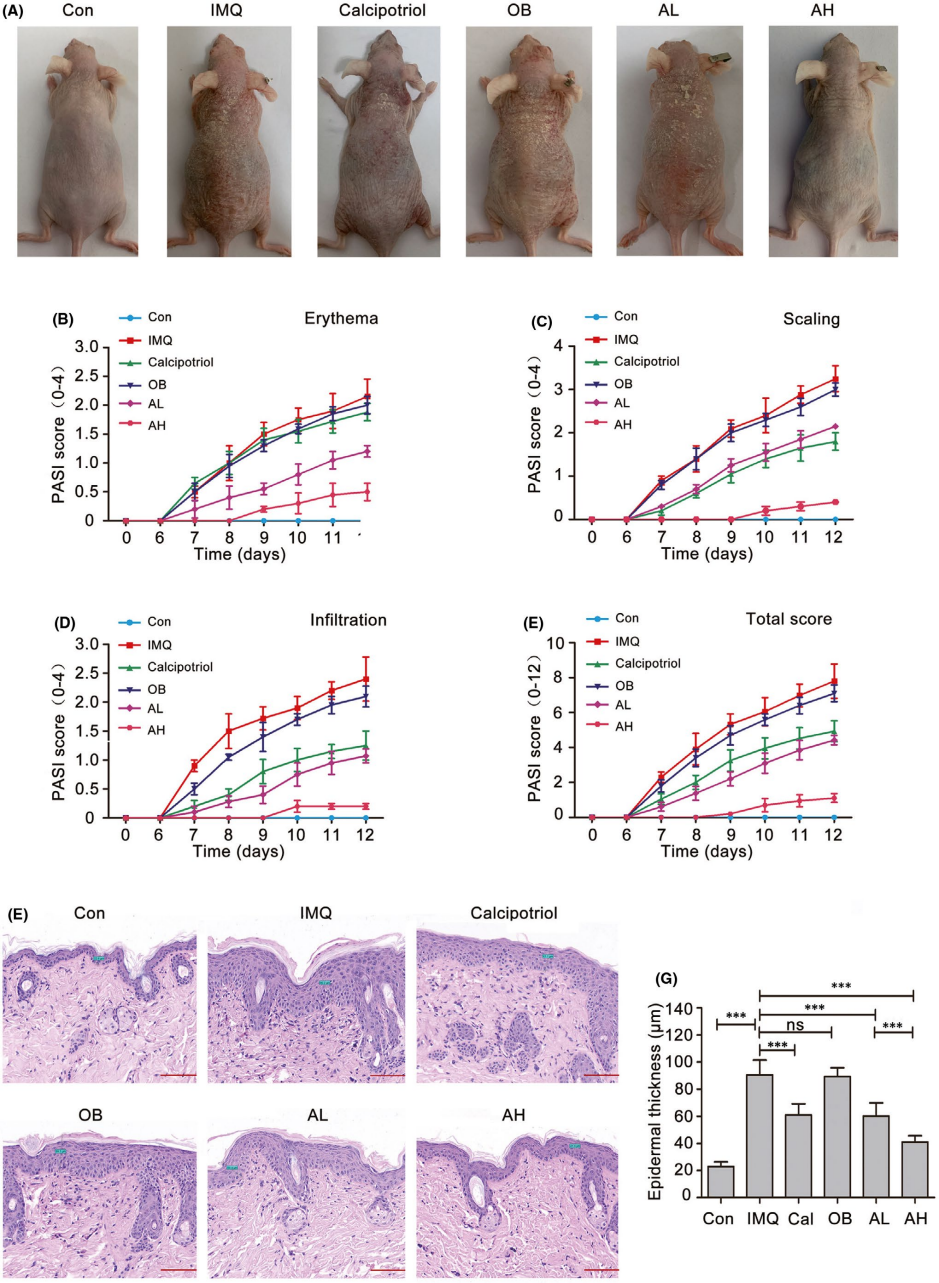
Astilbin treatment significantly attenuated IMQ‐induced psoriasis‐like lesions in mice in a dose‐dependent manner. SKH‐1 mice were treated daily with IMQ or control cream on the back skin for 12 days. On Day 6, IMQ‐treated mice were randomized into five groups and topically apply calcipotriol ointment, ointment bases (OB), 0.2% astilbin ointment (AL) or 0.5% astilbin ointment (AH) were applied topically after IMQ application for 0.5 h. (A) Representative back skin from the six groups is shown. (B–E) Psoriasis Area Severity Index (PASI) score of back skin lesions in the four groups including erythema, scaling and infiltration is scored daily on a scale from 0 to 4, and the total score is calculated. Data are represented as the mean ± SEM. (*n* = 5). (F) Representative haematoxylin and eosin staining (×200) analyses of mouse back skin from the different treatment groups and epidermal thickness are measured using CaseViewer 2.1 software (scale bar, 100 μm). (G) The epidermal thickness of the different treatment groups was statistically analysed. Data are represented as the mean ± SEM (*n* = 5), and one‐way ANOVA tests were used for comparison of the significant differences (***p < 0.001)

We first investigated the effect of astilbin on an IMQ‐induced psoriatic mouse model. IMQ cream was applied to the back skin of SKH‐1 mice for 12 consecutive days with or without calcipotriol ointment or different concentrations of astilbin cream. Typical erythema, scaling and infiltration were observed and recorded during the experiment, and the PASI score was used to evaluate the severity of psoriasis lesions. As shown in Figure 1A–E, on Days 6–8 after IMQ treatment, the back skin of the IMQ‐treated group began to show signs of psoriasis‐like skin lesions, such as erythema, scaling and thickening and the situation worsened over time. On Day 12, typical erythema and silvery white scales appeared in the IMQ‐treated mice, whereas calcipotriol‐treated mice had shallower erythema and sparser scales, while astilbin‐treated animals had less skin thickening, without visible erythema or scales (Figure 1A). The severity scores of psoriasis‐like symptoms in calcipotriol‐/astilbin‐treated animals appeared later and were significantly milder than those in the group treated with IMQ alone. In addition, the severity of psoriasis‐like skin lesions gradually decreased with the increasing of astilbin concentration.

Haematoxylin and eosin staining in Figure 1F shows that pathological changes in IMQ‐treated skin lesions included hyperkeratosis and parakeratosis of the epidermis, ‘Munro abscesses’ (composed of neutrophils) in the parakeratosis area, a thinned granular layer and thickened spinous layer, epidermal sudden downward extension of in‐depth dermis, capillary dilatation and hyperaemia and inflammatory cell infiltration in dermal papilla, a typical pathological phenotype of human psoriatic skin lesions. Astilbin significantly reduced epidermal thickness and attenuated IMQ‐induced psoriasis. Topical treatment with astilbin ointment clearly ameliorated the psoriasis‐like pathological changes induced by IMQ, evidenced by normal keratinization, reduced thickness of the epidermal layer (60.2 ± 9.6 μm in the AL group and 41.0 ± 4.6 μm in the AH group vs. 90.6 ± 10.8 μm in the IMQ group) and less inflammatory cell infiltration in skin lesions (Figure 1F,G). The therapeutic effect of astilbin was comparable with that of calcipotriol, an effective therapeutic drug for psoriasis treatment, with epidermal thinning, diminished stratum corneum and changes in skin compartments. These data demonstrated that astilbin topical treatment ameliorated the clinical and histological features of IMQ‐induced psoriasis‐like skin lesions in a dose‐dependent manner.

### 3.2 Topical astilbin reduced proliferation and promoted differentiation of keratinocytes in IMQ‐induced psoriasis‐like skin lesions

**FIGURE 2:**
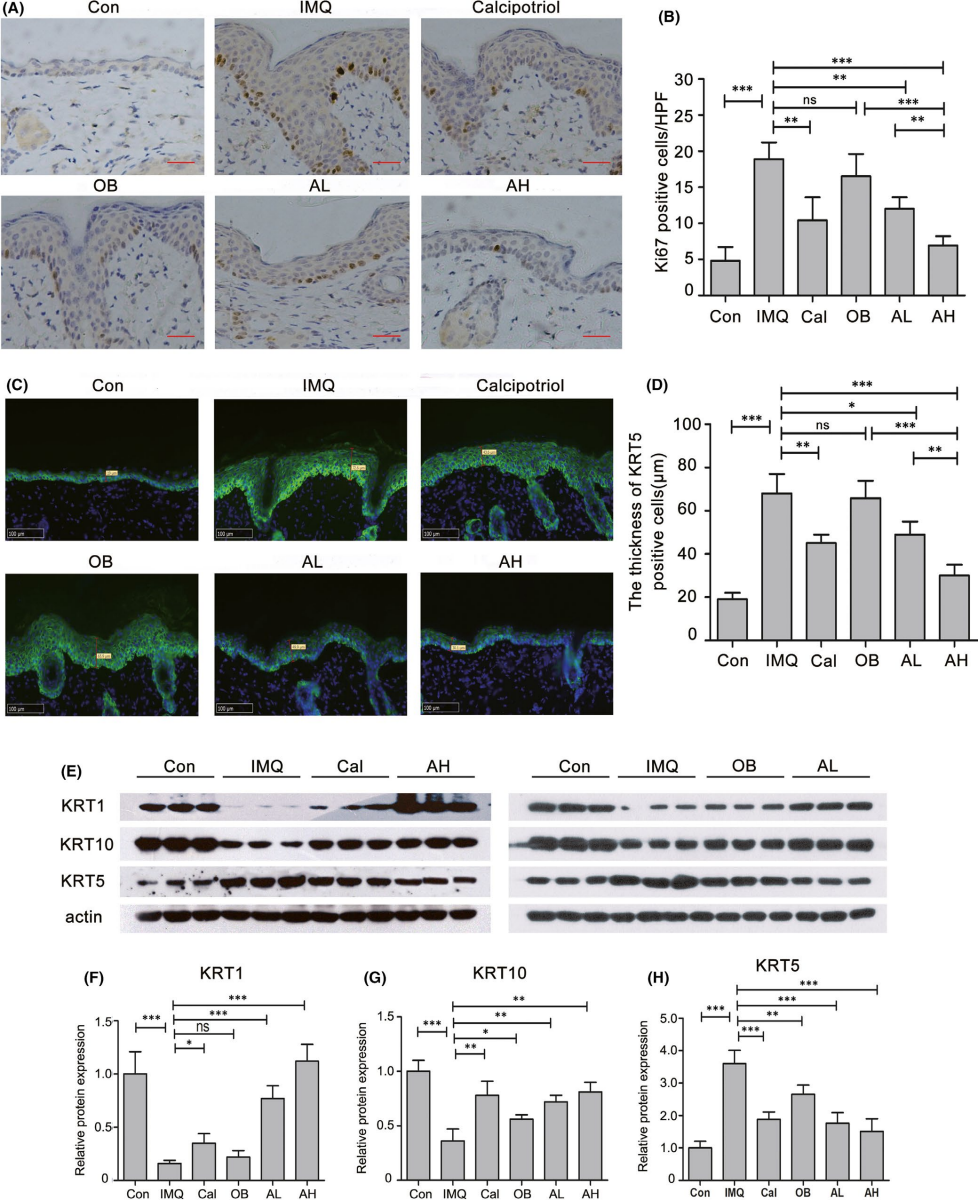
Astilbin topical treatment reduced proliferation and ameliorated differentiation of keratinocytes in IMQ‐induced psoriasis‐like skin lesions. (A) Immunohistochemical staining (×100) for Ki67 in mouse back skin of the different treatment groups (scale bar, 50 μm). (B) The mean number of Ki67‐positive cells ± SD in five representative high–power‐fields of the epidermis of individual mice with indicated treatment. (**p < 0.01, ***p < 0.001). (C) Immunofluorescence staining (×200) shows the change of KRT5 expression (green) in the epidermis from mouse back skin lesions with different treatment. (D) The thickness of KRT5‐positive cells in the epidermis of the different treatment groups was statistically analysed. Data are represented as the mean ± SEM (*n* = 5, *p < 0.05, **p < 0.01, ***p < 0.001). (E–H) Changes in expression levels of KRT1, KRT10 and KRT5 in mouse back skin from the different treatment groups were estimated by Western blotting analysis; actin served as a loading control. Protein amount was normalized to the amount of actin and was quantified by densitometry using Image J software. Results are the mean ± SD of three independent experiments (**p < 0.01, ***p < 0.001)

The epidermis is mainly composed of keratinocytes that undergo a gradual process of differentiation from basal to supra‐basal layers. The balance between proliferation and differentiation of keratinocytes is the basis of maintaining normal epidermal function. Ki67 expression in proliferative cells and Ki67 staining is a useful measure to evaluate the proliferative potential of epidermal keratinocytes. IMQ treatment increased epidermal thickness due to the hyperproliferation of basal keratinocytes, as indicated by the increased number of Ki67‐positive cells. However, topical astilbin significantly decreased Ki67‐positive keratinocytes in IMQ‐treated skin in a dose‐dependent manner (Figure 2A,B).

In the epidermal basal layer, keratinocytes express keratin 5 (KRT5) and KRT14; whereas in the spinous (supra‐basal) layers, KRT5/14 is progressively downregulated and replaced by KRT1/10. Thus, the expression pattern of keratins indicates the degree of differentiation.^20^ KRT5, a marker of undifferentiated keratinocytes, clearly increased in the epidermis of IMQ‐treated mice, as indicated by the thickness of KRT5‐positive cells reaching nearly 70 μm compared with 20 μm in the control mice (Figure 2C,D). However, calcipotriol and astilbin reversed the disrupted terminal differentiation of keratinocytes induced by IMQ. It is worth mentioning that in the epidermis of astilbin and IMQ‐treated mice, KRT5 staining gradually weakened from the basal layer to the stratum corneum (Figure 2C,D). Immunoblotting further confirmed that astilbin decreased KRT5 expression and increased the expression of KRT1 and KRT10 in a dose‐dependent manner, indicating differentiation of the epidermal keratinocytes (Figure 2E–H). These data suggest that topical administration of astilbin reduced IMQ‐induced proliferation of keratinocytes and effectively ameliorated IMQ‐induced keratinocyte dedifferentiation.

### 3.3 Topical astilbin decreased IMQ‐induced skin inflammation in mice

**FIGURE 3:**
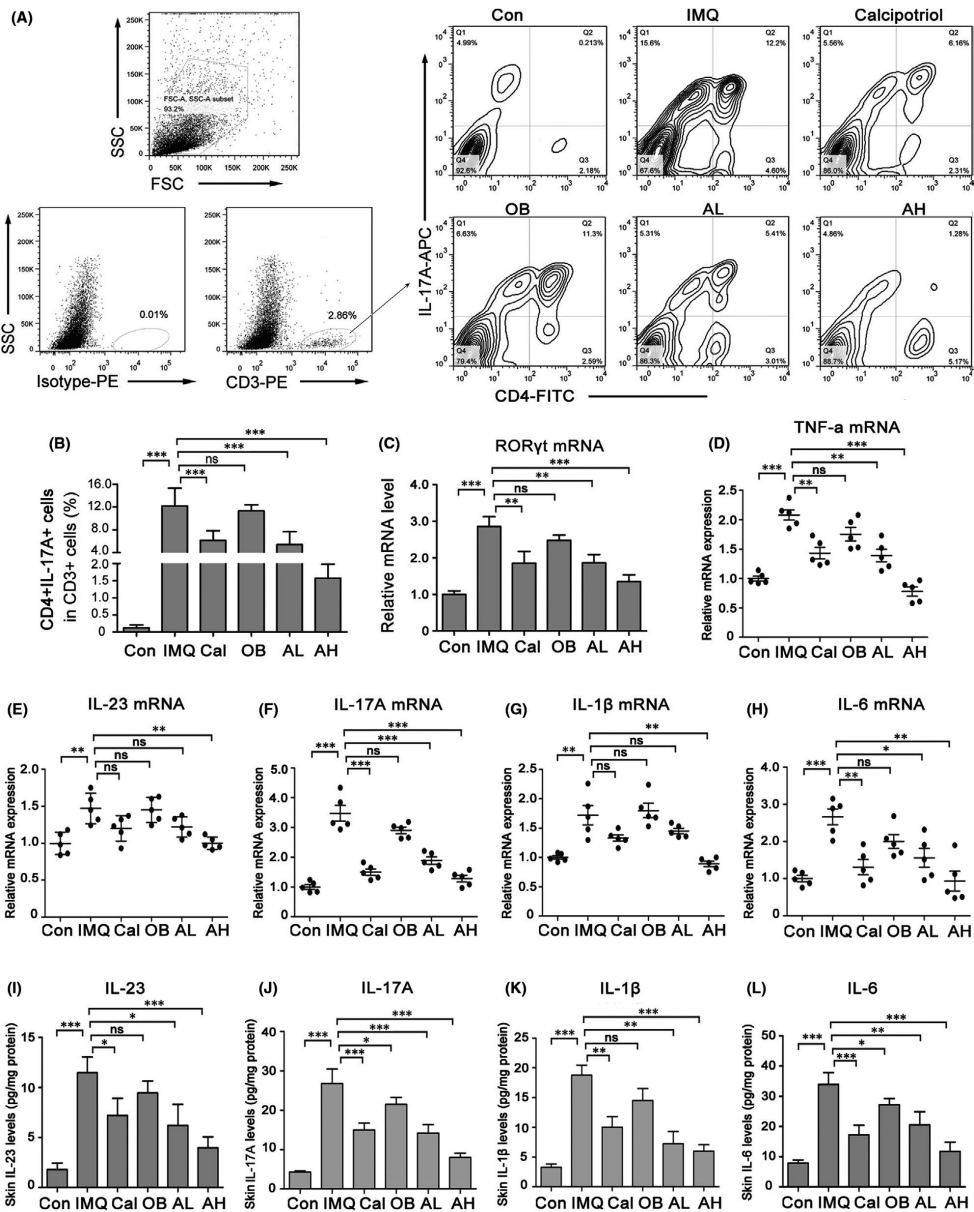
Topical astilbin decreased IMQ‐induced Th17 cell differentiation and skin inflammation in mice in a dose‐dependent manner. (A) Representative images of flow plot showing staining for CD4+IL‐17A+ T cell expression in CD3+ cells of mouse back skin in different groups. (B) The rate of CD4+IL‐17A+ T cell expression in CD3+ cells was analysed statistically. Data are expressed as the mean ± SEM (*n* = 5). (C) RORγt mRNA expression in IMQ‐induced skin lesions. (D–H) RT‐PCR for relative expression of TNF‐α, interleukin (IL)‐23, IL‐17A, IL‐1β and IL‐6 mRNAs in the different treatment groups. Values are shown as mean ± SEM, *n* = 5 (*p < 0.05, **p < 0.01 and ***p < 0.001 vs. IMQ group). (IL) ELISA was performed to assess the concentration of IL‐23, IL‐17A, IL‐1β and IL‐6 in mouse back skin of different groups. Data are represented as the mean ± SEM. (*n* = 5, *p < 0.05; **p < 0.01; *** *p* < 0.001)

The IL‐23/Th17 axis plays a role in the pathology of psoriasis.^6^ To investigate the effect of topical astilbin on IL‐17‐producing T cells, CD4^+^IL‐17A^+^ T cells in CD3^+^ cells were investigated by flow cytometry. As shown in Figure 3A,B, in CD3^+^ cells of IMQ‐induced psoriasis‐like skin lesions, CD4^+^IL‐17A^+^ T cells were significantly increased (12.760 ± 3.12% vs. 0.250 ± 0.082% in control skin lesions); while in astilbin‐treated mice, IL‐17A^+^ cells were significantly reduced in a dose‐dependent manner (5.410 ± 2.260% in the AL group and 1.575 ± 0.410% in the AH group). In addition, the mRNA level of the transcription factor RORγt, which specifically induces Th17 cell differentiation was reduced (Figure 3C).

The mRNA levels of psoriasis‐related inflammatory factors such as TNF‐α, IL‐23, IL‐17A, IL‐1β and IL‐6 were measured by RT‐PCR. Figure 3D–H shows that TNF‐α, IL‐23, IL‐17A, IL‐1β and IL‐6 were significantly suppressed after astilbin treatment in the IMQ‐induced mouse skin. ELISA results (Figure 3G–I) showed that astilbin inhibited IMQ‐induced IL‐23, IL‐17A, IL‐1β and IL‐6 protein expression on different degrees. These results suggest that topical astilbin can inhibit the differentiation of Th17 cells and IMQ‐induced skin inflammation.

### 3.4 Astilbin inhibited the maturation and activation of BMDCs and decreased the expression of pro‐inflammatory cytokines

**FIGURE 4:**
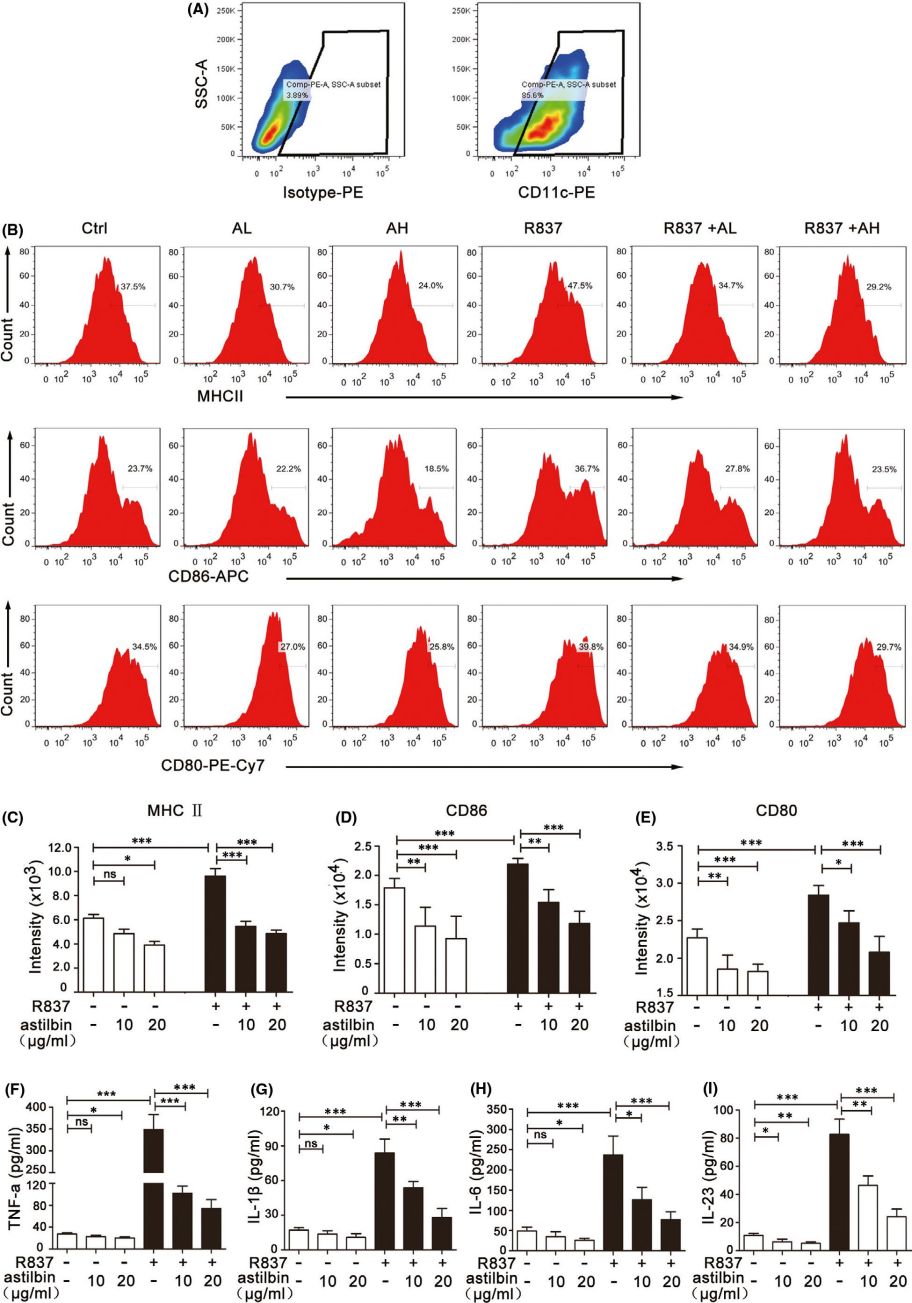
Astilbin inhibits R837‐induced maturation and activation of bone marrow‐derived dendritic cells (BMDCs). (A) Gating strategy for CD11c+ BMDCs (gate on the right). BMDCs were stained with CD11c and isotype control mAbs to reveal CD11c^+^ cells. (B) Flow cytometry was performed to analyse the expression change of MHCⅡ/CD86/CD80 in BMDCs that were exposed to R837 and/or different concentration of astilbin. (C–E) The rate of BMDCs expressing MHCⅡ/CD86/CD80 stimulated by R837 and/or different concentration of astilbin was statistically analysed. Data are expressed as the mean ± SD (*n* = 3). *p < 0.05, **p < 0.01 and ***p < 0.001. (F–I) ELISA was performed to assess the concentration of TNF‐α, IL‐1β, IL‐6 and IL‐23 in the supernatant of R837‐induced BMDCs pretreated or not with astilbin (10 or 20 μg/ml, 6 h). The results are shown as the mean ± SD of three separate experiments (*p < 0.05; **p < 0.01; ***p < 0.001)

It is well known that mature dendritic cells (mDCs) affect the maturation and function of Th17 cells; so, to further determine the mechanism underlying the inhibition of IL‐17‐producing T cells and inflammatory cytokine expression by astilbin, we investigated the effects of astilbin on maturation and activation of DCs in BMDCs stimulated with R837(IMQ). First, DCs were screened by CD11c‐PE staining after BMDCs were cultured with IL‐4 and GM‐CSF for 7 days and then stimulated with astilbin and/or R837 (Figure 4A). Later, the DC maturation markers MHCII, CD80 and CD86 were examined by flow cytometry. At the same time of detection, the levels of TNF‐α, IL‐23, IL‐1β and IL‐6 in the culture medium were detected by ELISA.

**FIGURE 5:**
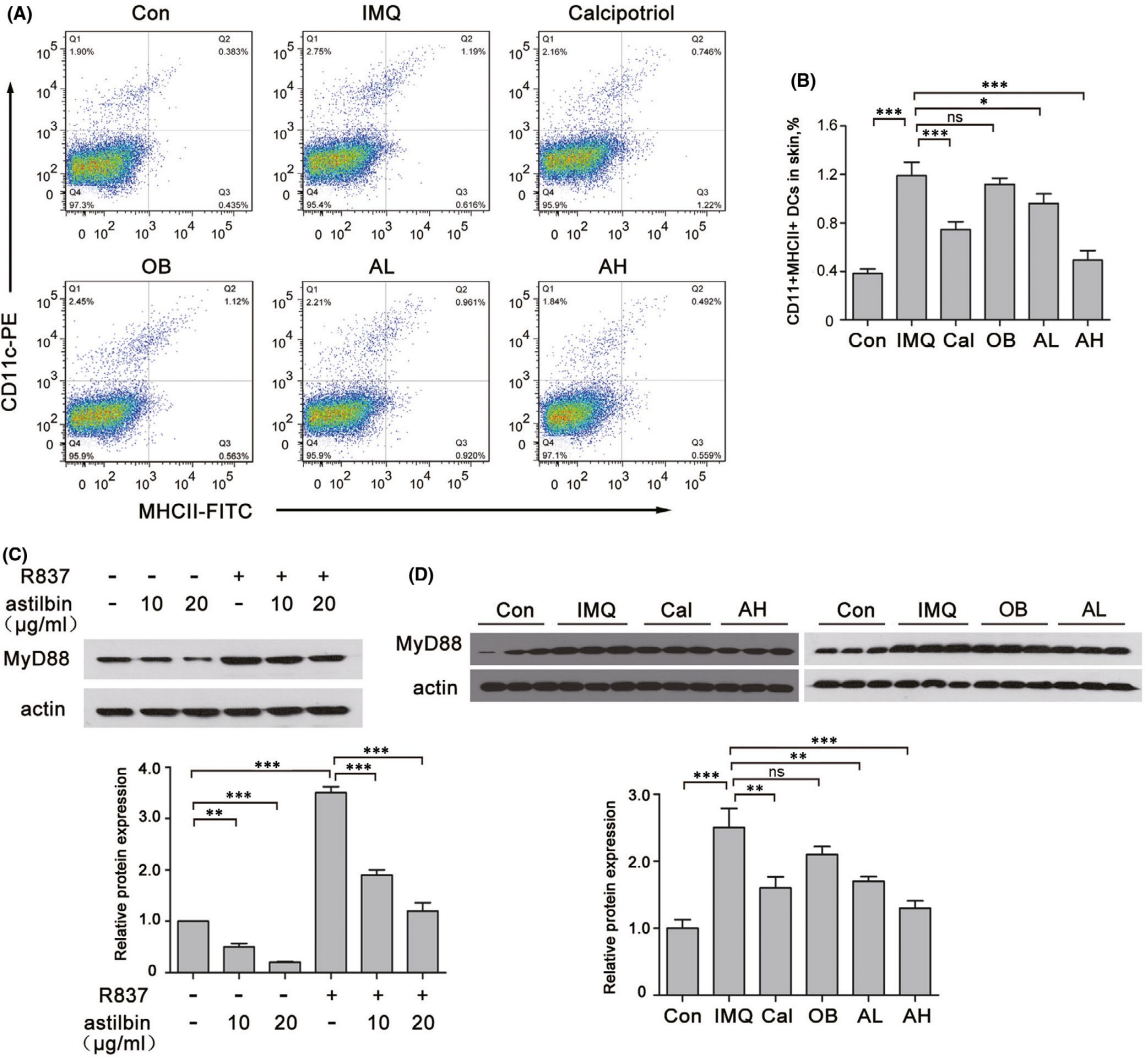
Topical astilbin decreased IMQ‐induced mDCs in mice in a dose‐dependent manner. (A) Flow cytometry was performed to analyse the expression change of CD11+MHCⅡ+ DCs of mouse back skin in the different groups. (B) The rate of CD11+MHCⅡ+ DCs expression was statistically analysed. Data are expressed as the mean ± SEM (*n* = 5, **p < 0.01 and ***p < 0.001). (C) Western blot analysis of MyD88 expression in R837‐induced BMDCs pretreated or not with astilbin. Actin was used as a loading control. Protein amounts were normalized to the amount of actin and were quantified using the densitometry function of ImageJ software. The results are the mean ± SD of three independent experiments. **p < 0.01, ***p <0.001. (D) The levels of MyD88 in skin lesions of the different treatment groups were assessed by Western blotting. Actin was used as a loading control. Protein amounts were normalized to the amount of actin. The results are the mean ± SD of three independent experiments. (**p < 0.01, ***p < 0.001)

Flow cytometry analysis revealed that R837 dramatically increased the expression of MHCII, CD80 and CD86, while astilbin decreased the expression of MHCII, CD80 and CD86 stimulated by R837 to various degrees (Figure 4B–E). Astilbin at 20 μg/ml significantly inhibited R837‐induced TNF‐α, IL‐23, IL‐1β and IL‐6 protein expression in BMDCs (Figure 4F–I), especially IL‐6, which was significantly different from the control. In addition, the change in DCs was detected by flow cytometry in vivo (Figure 5A,B). After treatment, the skin lesions were collected and digested into single cells to analyse CD11c^+^MHCII^+^ DCs. In IMQ‐induced psoriasis‐like skin lesions, CD11c^+^MHCII^+^ DCs were significantly increased (1.19 ± 0.11% vs. 0.38 ± 0.04% in control skin lesions); while in astilbin‐treated mice, CD11c^+^MHCⅡ^+^ DCs were significantly reduced in a dose‐dependent manner (0.96 ± 0.078% in the AL group and 0.492 ± 0.08% in the AH group).

We further explored whether astilbin inhibits the activation of TLR7/8 signalling by detecting MyD88. The results (Figure 5C) showed that astilbin significantly prevented the overexpression of MyD88 stimulated by R837 in DCs. In addition, lower protein expression levels of MyD88 were observed in skin lesions of the astilbin‐treated group as compared to those levels in the IMQ‐induced model (Figure 5D).

## 4 DISCUSSION

Astilbin is the predominant bioactive component extracted from the Chinese medicinal herb—Rhizome smilacis glabrae, which is traditionally used to treat inflammatory diseases.^21^ In recent years, astilbin has attracted considerable attention for its antioxidant and anti‐inflammatory effects,^22^, ^23^ with no hepatotoxicity, renal toxicity or genotoxicity.^24^ Our previous study^14^ and the study by Li et al.^13^ have reported that astilbin has an anti‐proliferative effect on keratinocytes in vitro and anti‐differentiation effects on Th17 cells. Our study further demonstrated that topical administration of astilbin at a lower dose level in an IMQ‐induced psoriasis‐like murine model improves psoriasis‐like inflammation and scales in mice. This is largely due to the astilbin‐induced decrease in mature DCs stimulated by the TLR7/8 agonist, subsequent inhibition of Th17 /IL‐17A‐induced immune responses and excessive keratinocyte proliferation. In this study, we chose SKH‐1 mice because they do not need to be shaved to establish the psoriasis model; therefore, skin lesions are easily observed. Notably, we used IMQ for 12 days to induce psoriasis‐like skin lesions in SKH‐1 mice, although the common treatment time in female BALB/c mice is 7 days. The different application times of IMQ are due to the use of different strains of mice. The mechanisms underlying the different application times of IMQ are unclear, as less hair may result in lower degrees of drug absorption, or it may be due to the lack of the physical stimulation of shaving.

Psoriasis is a common chronic recurrent inflammatory skin disease mediated by immune cells and molecules, with persistent epidermal hyperplasia. The IL‐23/Th17 axis has been shown to play a role in the pathology of psoriasis, and drugs targeting the IL‐23/Th17 axis show promising efficacy in clinical trials with favourable side effect profiles.^25^ Differentiation from Th0 to Th17 cells depends on signals and pro‐inflammatory cytokines such as IL‐6/IL‐23 to be released from mDCs with cell surface proteins like MHCII/CD80/CD86.^26^ Therefore, strategies that block the maturation of DCs and the release of pro‐inflammatory cytokines from DCs may be a promising approach against psoriasis. Our study revealed that astilbin decreased the expression of MHCII, CD80 and CD86 stimulated by R837 to various degrees and significantly inhibited R837‐induced TNF‐α, IL‐23, IL‐1β and IL‐6 protein expression in BMDCs. However, activation of the DCs/IL‐23/Th17 signal in psoriasis is not entirely clear. In recent years, research on psoriasis pathogenesis has largely expanded our knowledge of the skin microbiome; one of the proposed mechanisms is that antimicrobial peptides (AMPs) secreted by keratinocytes in response to bacterial/viral infections or injury can bind to self‐DNA/RNA fragments released from affected keratinocytes to stimulate pDCs through TLR7/8,^27^ then release high amounts of TNF‐α, IL‐12 and IL‐23.^28^ One of the most relevant AMPs to the pathology of psoriasis is cathelicidin (LL37). IMQ (R837), a ligand of TLR7/8, can activate TLR7 to recruit adaptor protein MyD88, which subsequently forms a complex with other downstream molecules (IRAK4, TRAF6, IRAK1 and IRAF7). This complex can activate NF‐κB and phosphorylate IRF7. Activated NF‐κB and phosphorylated IRF7 are then translocated into the nucleus to induce the expression of pro‐inflammatory cytokines and type I interferons.^29^ MHC II, CD80 and CD86 are also induced in a MyD88‐dependent manner in the TLR7 signalling pathway.^30^ Therefore, MyD88‐related signalling is the principal signal transduction pathway of TLR7/8. In this study, the expression of MyD88 was significantly increased in IMQ‐induced mice, while astilbin significantly inhibited MyD88 expression as compared to calcipotriol. Thus, astilbin plays an anti‐inflammatory role in psoriasis by inhibiting maturation and activation of DCs, probably through the TLR7/MyD88 signalling pathway.

In conclusion, previous studies have reported that oral astilbin could inhibit Th17 cell differentiation and ameliorate IMQ‐induced psoriasis‐like skin lesions by inhibiting the Jak3/Stat3 signalling pathway in BALB/c mice.^13^ Furthermore, astilbin possesses anti‐proliferative and differentiation‐modulating effects on keratinocytes in vitro. This study revealed that topical administration of astilbin at a lower dose targeted the TLR7/8 signalling pathway, subsequently inhibiting Th17/IL‐17A‐induced immune responses and excessive keratinocyte proliferation to ameliorate IMQ‐induced psoriasis‐like skin lesions in SKH‐1 mice. Therefore, topical administration of astilbin may be a promising agent for psoriasis treatment, and appropriate dosage forms for external use are under development.

## CONFLICT OF INTEREST

The authors declare that there are no conflicts of interest.

## AUTHOR CONTRIBUTIONS

**Qingqing Xu:** Data curation (lead); formal analysis (lead); supervision (lead); writing – original draft (lead). **Zhaoyang Liu:** Data curation (equal); methodology (equal). **Zhiqiang Cao:** Conceptualization (equal); methodology (equal); software (equal). **Ning Yang:** Conceptualization (equal); methodology (equal). **Yongjian Shi:** Software (equal); supervision (equal); validation (equal). **Chunmin Zhang:** Software (equal); supervision (equal); validation (equal); writing – original draft (equal). **Chunhong Zhang:** Funding acquisition (lead); methodology (lead); supervision (lead); writing – review and editing (lead). **Guangshang Cao:** Project administration (equal). **Rong Sun:** Writing – original draft (equal).

## Data Availability

The data that support the findings of this study are available from the corresponding author upon reasonable request.
